# Real-Time Integration of an AI-Based ECG Interpretation System in the Emergency Department: A Pragmatic Alternating-Day Study of Diagnostic Performance and Clinical Process Metrics

**DOI:** 10.3390/healthcare14070968

**Published:** 2026-04-07

**Authors:** Min Seok Choi, Su Il Kim, Yun Deok Jang, Seong Ju Kim, In Hye Kang, Woong Bin Jeong

**Affiliations:** 1 Department of Health Administration, Yeungnam University College, Daegu 42415, Republic of Korea; dreebok@naver.com; 2 Department of Paramedicine, Yeungjin University, Daegu 41527, Republic of Korea; ksi5328@yju.ac.kr; 3 Department of Paramedicine, Tongmyong University, Busan 48520, Republic of Korea; jangyundeok@naver.com (Y.D.J.); superemt@tu.ac.kr (S.J.K.); 4 Department of Paramedicine, Daewon University College, Jecheon 27135, Republic of Korea; emtkih@daewon.ac.kr; 5 Emergency Medicine Department, Inje University Busan Paik Hospital, Busan 47392, Republic of Korea

**Keywords:** artificial intelligence, electrocardiogram, emergency department, ST-elevation myocardial infarction, diagnostic performance

## Abstract

**Background/Objectives:** Rapid and accurate electrocardiogram (ECG) interpretation is essential for timely recognition of ST-elevation myocardial infarction (STEMI) and initiation of reperfusion therapy in the emergency department (ED). We evaluated the diagnostic performance of a real-time artificial intelligence (AI) ECG interpretation system and its pragmatic impact when integrated into routine ED workflows. **Methods:** This prospective, single-center pragmatic observational study was conducted in a regional emergency medical center ED in Busan, Republic of Korea (1 January–31 December 2024). Consecutive adults (≥18 years) undergoing 12-lead ECG for cardiovascular-related symptoms were enrolled (N = 1524). A predefined alternating-day protocol allocated visits to physician-only interpretation days (physician-days, N = 763) or AI output disclosure days (AI-days, N = 761). Diagnostic performance for STEMI was assessed using paired ECG-level comparisons between physician-alone interpretation and AI output against a blinded expert-panel reference standard; clinical impact outcomes included reperfusion-related time metrics, hospital length of stay (LOS), and in-hospital mortality. **Results:** Against the expert reference standard, AI showed higher STEMI sensitivity than physician-alone interpretation (96.7% vs. 68.3%; McNemar *p* = 0.027), while specificity was lower (75.9% vs. 84.5%; *p* = 0.018). In pragmatic day-level comparisons, door-to-balloon time was shorter on AI-days (40.0 ± 19.81 vs. 47.34 ± 21.90 min; *p* = 0.001), and time to PCI was significantly reduced among patients with atypical presentations (42.3 ± 18.21 vs. 57.1 ± 20.11 min; *p* = 0.013). Among admitted patients, hospital LOS was shorter on AI-days (13 ± 9.21 vs. 17 ± 10.31 days; *p* = 0.010), whereas in-hospital mortality did not differ significantly between groups (17.0% vs. 16.77%; *p* = 0.191). **Conclusions:** Real-time AI-ECG integration in the ED was associated with improved STEMI detection sensitivity and shorter reperfusion-related time metrics, particularly in atypical presentations, and with reduced hospital LOS among admitted patients. Short-term mortality was comparable between groups. Further multicenter studies are warranted to confirm generalizability and to balance benefits against potential false-positive-related operational impacts.

## 1. Introduction

Electrocardiography (ECG) performed in the emergency department (ED) is an essential diagnostic tool for the early identification of acute cardiovascular diseases and can also provide important clues for systemic conditions such as electrolyte disturbances, drug toxicity, and respiratory disorders [1]. In patients presenting with chest pain, palpitations, or dyspnea, rapid ECG-based evaluation plays a pivotal role in the early detection and management of acute coronary syndrome (ACS) [2]. Current guidelines recommend obtaining and interpreting a 12-lead ECG within 10 min of ED presentation for patients with suspected ACS [3], underscoring the clinical value of ECG as a frontline screening and decision-support test in time-sensitive cardiovascular emergencies [4].

Beyond the ED, prehospital care increasingly emphasizes early assessment, enabling trained providers to acquire 12-lead ECGs in the field and share results with receiving hospitals to facilitate early identification of ST-elevation myocardial infarction (STEMI) and streamline preparation for reperfusion therapy within guideline-recommended time windows [5,6,7]. Nevertheless, reliance on symptoms and clinical signs alone has notable limitations in accurately identifying acute cardiovascular conditions, particularly among patients with atypical presentations, reinforcing the need for rapid ECG acquisition and interpretation in emergency care since its introduction in the early 20th century [8,9,10].

**Figure 1:**
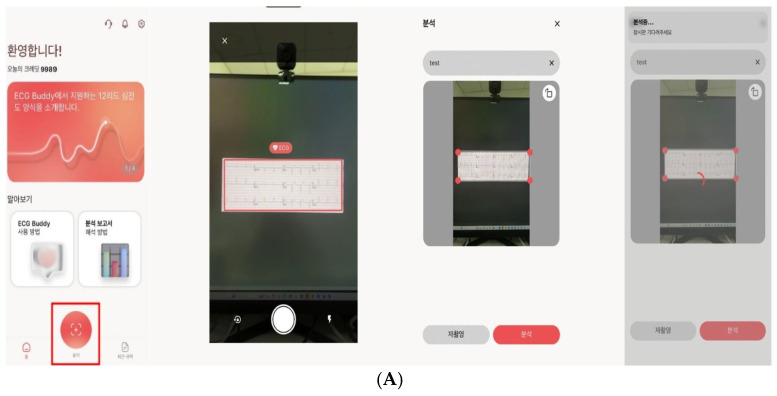

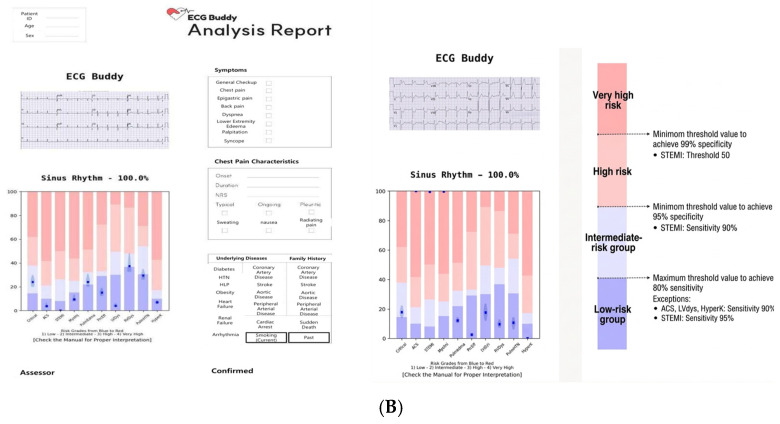
(**A**) Workflow of the ECG Buddy Analyzer. The application captures a photographed ECG, processes the image using a deep learning convolutional neural network, and displays the analysis results within approximately 10 s. Higher-resolution images and clearer labeling have been applied to improve readability. (**B**) Record sheet for emergency situations and cardiac-dysfunction risk assessment. It is capable of classifying 35 different types of cardiac rhythms. One of its core features is the stratification of cardiac dysfunction risk into four categories: very high risk, high risk, intermediate risk, and low risk.

With the rapid development of artificial intelligence (AI), deep learning-based algorithms capable of learning complex patterns from large-scale datasets have been proposed as tools to reduce interpretation errors and support clinical decision-making [11,12,13]. Recent advances in deep learning have enabled more sophisticated signal processing approaches, including multichannel feature fusion techniques for cardiovascular signal analysis, further improving classification performance and robustness [14]. In Korea, an AI-based ECG analysis program has been developed using deep learning technology that can analyze ECG images and provide an automated interpretation; importantly, in ED settings, the system can be implemented to generate outputs immediately after ECG acquisition and display them to clinicians in real time (Figure 1).

Against this backdrop, the present study evaluates the diagnostic performance of an AI-based ECG interpretation system and examines its pragmatic impact when integrated into real-world ED workflows. Although prior work suggests that AI-assisted ECG analysis may improve detection of ischemia and other acute conditions, evidence remains limited regarding real-time workflow integration and downstream clinical processes—such as time to intervention, length of stay, and short-term outcomes—across broad ED populations [15].

This study had three objectives: (1) to assess diagnostic concordance between emergency physicians and an AI-based ECG analysis system among adult ED patients presenting with cardiovascular symptoms; (2) to evaluate the association of early AI-assisted ECG interpretation with key clinical process metrics and short-term outcomes among patients diagnosed with myocardial infarction; and (3) to provide empirical evidence on the feasibility and potential clinical impact of integrating AI-driven ECG decision-support tools into emergency care.

## 2. Materials and Methods

This study was designed as a prospective, pragmatic observational investigation with a retrospective analytical component. The study period (1 January to 31 December 2024) refers to the timeframe during which ECG data were generated and stored in the hospital database as part of routine clinical practice, rather than the period of research conduct. The study evaluated the diagnostic performance and clinical impact of integrating a real-time artificial intelligence (AI) electrocardiogram (ECG) interpretation system (ECG Buddy Analyzer, South Korea) into routine ED care. The manuscript is reported in accordance with STROBE and the AI-specific reporting guidance (CONSORT-AI extension and DECIDE-AI).

### 2.1. Clinical Workflow and Exposure Protocol

**Figure 2:**
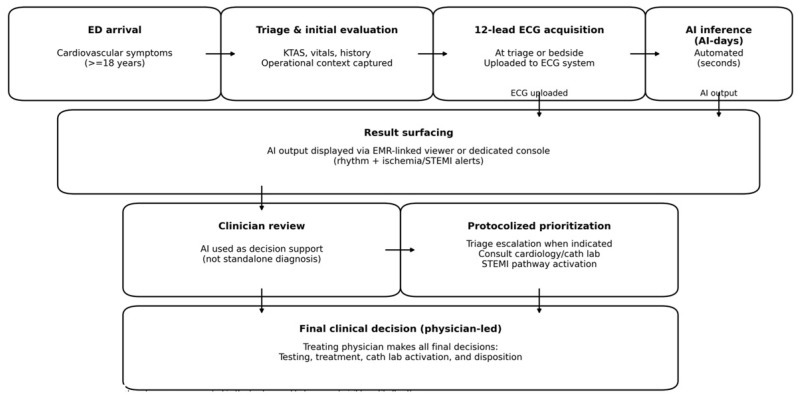
Clinical workflow integration of real-time AI-ECG interpretation on AI-days.

A predefined alternating-day exposure protocol was applied at the ED level. On physician-exposure days (“physician-days”), 12-lead ECGs were interpreted according to usual care by the treating emergency physicians, without real-time AI display. On AI-exposure days (“AI-days”), AI outputs were automatically generated immediately after ECG acquisition and displayed to the treating team in real time via the site’s designated viewing interface as a decision-support tool. In both conditions, the attending physician retained full responsibility for all clinical decisions, including triage escalation, diagnostic testing, activation of STEMI pathways, catheterization laboratory consultation, and disposition. The study did not mandate any specific actions in response to AI outputs. The end-to-end workflow for ECG acquisition, AI inference, and result surfacing is summarized in Figure 2.

To minimize contamination, on physician-days the AI system performed background analyses for study purposes, but outputs were not visible to clinicians until after the initial physician interpretation had been documented in the medical record. Clinicians received standardized training on the interface and were instructed to use AI outputs as decision-support rather than as a standalone diagnosis. The emergency department was staffed by board-certified emergency physicians, with trainee support under attending supervision, consistent with routine practice. ECG interpretation and STEMI pathway activation followed standard institutional protocols, and clinicians received a standardized orientation to the AI interface prior to study initiation, emphasizing that AI output was used as an adjunct to clinical judgment. And given the pragmatic alternating-day allocation strategy, exposure was not randomized. Therefore, in addition to patient-level covariates, our models adjusted for prespecified temporal factors including shift category based on ED arrival time, weekday versus weekend, and calendar month, and we conducted sensitivity analyses stratified by shift and weekday versus weekend to assess the robustness of the findings (Table A1, Table A2 and Table A3). The alternating-day design was selected to enable pragmatic real-world implementation while minimizing workflow disruption and contamination between exposure conditions. More complex designs, such as cluster randomization or stepped-wedge approaches, were considered but were not feasible within the operational constraints of the emergency department.

### 2.2. Study Setting and Participants

**Figure 3:**
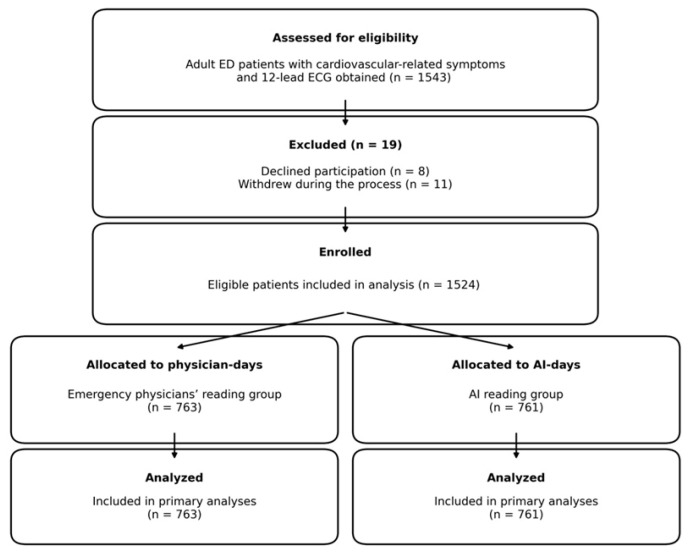
Study flow diagram. A total of 1543 adult patients presenting to the emergency department with cardiovascular-related symptoms were screened. After excluding 19 patients (8 declined to participate and 11 withdrew during the process), 1524 patients were enrolled and allocated by a predefined alternating-day protocol to the emergency physicians’ reading group (N = 763) or the AI reading group (N = 761); all were included in the primary analyses.

The study site provides 24/7 cardiology consultation and on-site catheterization laboratory capability and serves as a major regional referral destination for suspected acute coronary syndrome (ACS). Eligible participants were consecutive adult patients (≥18 years) presenting to the ED with cardiovascular-related symptoms for whom a 12-lead ECG was obtained as part of routine care. In total, 1543 patients were screened. Patients were excluded if they declined participation or withdrew after screening (N = 19). The final analytic cohort comprised 1524 patients (Figure 3).

Symptom categories were defined a priori using the ED triage chief complaint and initial clinician documentation. Typical symptoms included chest pain/pressure and dyspnea, whereas atypical symptoms included epigastric discomfort, dizziness, syncope, and other presentations without chest pain, consistent with operational definitions used in ED chest pain pathways. Two independent physicians reviewed all index ECGs, blinded to allocation group and clinical outcomes, and assigned a reference diagnosis of STEMI versus non-STEMI. Disagreements were resolved through consensus discussion and, when necessary, adjudication by a third senior reviewer. Inter-expert agreement for the initial independent ratings was assessed using percent agreement and Cohen’s kappa with 95% confidence intervals. Reporting inter-expert agreement supports the validity and reproducibility of the expert-panel reference standard.

### 2.3. Ethics and Consent

Written informed consent was obtained whenever feasible. For critically ill patients unable to provide consent at presentation, consent was obtained from a legally authorized representative. When neither the patient nor a proxy was immediately available and time-sensitive care was required, enrollment proceeded under an institutional review board-approved emergency waiver/deferred consent process, and written consent was obtained as soon as practicable. Research staff supported enrollment procedures to avoid delaying emergency care. This study involved a retrospective analysis of existing clinical data. No data were collected for research purposes prior to IRB approval. The institutional review board approval was obtained on 5 December 2023, which permitted access to and analysis of previously recorded clinical data with a waiver or modification of informed consent where applicable (IRB No. 2023-11-051). The IRB approved both the pragmatic clinical implementation and the retrospective analysis of routinely collected clinical data.

All analyses involving identifiable patient data were conducted after IRB approval, in compliance with applicable ethical guidelines.

### 2.4. AI System and ECG Acquisition

Upon ED arrival, patients underwent standard triage and initial evaluation, and a 12-lead ECG was acquired per institutional protocol and uploaded to the hospital ECG management system. On AI-days, uploaded or photographed ECGs were analyzed by the AI engine within seconds, and interpretation outputs—including rhythm classification and ischemia/myocardial infarction alerts—were surfaced to the treating team through the designated clinical interface.

The AI-based electrocardiogram (ECG) interpretation system used in this study was ECG Buddy Analyzer (ARPI Inc., Seongnam, Republic of Korea), a deep learning-based decision-support software designed to assist real-time clinical decision-making by analyzing standard 12-lead ECGs, including ECG images captured at the point of care and/or digital ECG data. ECG Buddy is a software-as-a-medical-device intended to support clinicians in ECG interpretation (rather than replace clinician judgment) by providing automated rhythm interpretation and quantitative risk information relevant to emergency care and cardiac dysfunction. In routine use, the platform outputs automated rhythm-type classifications and quantitative ECG-derived digital biomarkers (risk scores) that summarize the likelihood of clinically important conditions. In the specific Analyzer configuration deployed in our emergency department workflow, the system was configured to support recognition of acute coronary syndrome and ST-segment elevation myocardial infarction (STEMI), to classify 35 rhythm categories, and to stratify cardiac dysfunction risk into four levels (very high, high, intermediate, and low), with results returned in real time for clinician review. Detailed information regarding the underlying training dataset, labeling procedures, and prior internal/external validation is not fully available in public-facing documentation; therefore, we report the system’s intended use, regulatory status, and output structure, and we emphasize that all diagnostic and treatment decisions remained the responsibility of the treating clinicians and that the system did not autonomously order tests or activate catheterization laboratory pathways.

The AI system used in this study is a commercially available, proprietary deep learning-based software. Detailed information on the training dataset, including sample size, demographic composition, labeling procedures, and external validation cohorts, is not publicly available due to proprietary restrictions.

However, the system is designed to analyze standard 12-lead ECG data and has been developed using supervised deep learning. We acknowledge that the lack of transparency in model development may limit the assessment of potential bias and its generalizability across different populations and clinical settings. Key deployment characteristics (automatic inference immediately following ECG acquisition, result surfacing via the designated viewer, and principles governing clinical exposure and use) are documented in Figure 1. The structured ECG response form used for reference standard labeling is provided in the Appendix A.

### 2.5. Reference Standard and Outcomes

#### 2.5.1. Diagnostic Performance Evaluation (Paired ECG-Level Analysis)

Diagnostic accuracy was evaluated using an ECG-level paired-comparison framework. For each ECG, two index assessments were defined: the treating emergency physician’s initial clinical interpretation documented in the medical record and the AI system output generated for the same ECG. These assessments were compared against a common reference standard derived from a blinded expert-panel consensus.

To preserve a “physician-alone” evaluation, the primary paired diagnostic performance analysis used ECGs from physician-days, during which AI outputs were generated in the background for study purposes but were not visible to clinicians until after physician documentation was completed.

The primary diagnostic endpoint for accuracy analysis was STEMI presence/absence. ECG evidence consistent with NSTEMI was evaluated as a secondary endpoint, acknowledging that definitive NSTEMI diagnosis may require biomarker and clinical information; therefore, NSTEMI findings are interpreted as ECG-based detection performance and are complemented by visit-level clinical outcome analyses.

Performance metrics (sensitivity, specificity, positive predictive value [PPV], and negative predictive value [NPV]) were calculated with exact (Clopper–Pearson) 95% confidence intervals (CIs). Because physician and AI classifications were available for the same ECGs, discordant paired classifications were compared using McNemar’s test (two-sided).

#### 2.5.2. Clinical Impact Evaluation (Pragmatic Day-Level Comparison)

Pragmatic clinical impact was evaluated at the visit level using the predefined alternating-day exposure protocol. On physician-days, ECG interpretation proceeded under usual care without real-time AI display, whereas on AI-days, AI outputs were generated immediately after ECG acquisition and displayed to the treating team as a decision-support tool; clinical decisions remained at the discretion of the attending physician. Prespecified visit-level outcomes included time to PCI, hospital length of stay, ICU admission, in-hospital mortality, and utilization outcomes (angiography/PCI) and disposition.

For clinical impact analyses, regression models compared AI-days versus physician-days with adjustment for baseline covariates (e.g., age, sex, comorbidity burden, KTAS, arrival mode, and initial vital signs) and operational covariates (hour-of-day, weekday/weekend, and calendar month), with additional adjustment for ED crowding surrogates when available. Because exposure assignment occurred by calendar day, cluster-robust standard errors at the day level were used to account for within-day correlation.

#### 2.5.3. Expert-Panel Reference Standard

The reference standard was derived from offline review by an expert panel comprising five board-certified emergency medicine specialists with substantial ECG interpretation experience (≥10 years post-board certification). Expert reviewers interpreted ECGs offline using only the ECG tracing and were blinded to study arm assignment, AI outputs, symptoms, vital signs, laboratory/imaging results, treatments, and clinical outcomes. Interpretations were recorded using a structured ECG response form (Appendix A) and included rhythm classification and the presence/absence of STEMI and ECG evidence consistent with NSTEMI. A consensus reference label was determined by majority vote (≥3 of 5 reviewers). A board-certified cardiologist (>10 years of clinical experience) adjudicated ECGs with discordant expert votes (no majority) and reviewed a random sample of majority-negative ECGs to assess internal consistency. Inter-rater reliability was summarized using Fleiss’ kappa, and the proportion of adjudicated changes was reported.

### 2.6. Data Collection and Variables

Data were collected via structured electronic-medical-record review using predefined variable definitions. Baseline variables included age, sex, arrival mode, triage acuity (Korean Triage and Acuity Scale, KTAS), initial vital signs, and comorbidity burden (Charlson Comorbidity Index, CCI). To capture operational confounding, visit timing (day/evening/night), day of week (weekday/weekend), and calendar month were extracted, along with contemporaneous ED crowding surrogates at ECG time when available (e.g., ED census and boarding count). Follow-up variables included final diagnoses, coronary angiography/PCI, admission, ICU admission, hospital length of stay, in-hospital mortality, and discharge status. Time to PCI was defined as the interval from ED arrival to initiation of PCI. Laboratory data (including CK-MB and troponin I) were extracted, and initial chest radiographs were reviewed for secondary signs of cardiac dysfunction when available.

### 2.7. Statistical Analysis

Statistical analyses were performed using IBM SPSS Statistics (version 27, IBM Corp., Armonk, NY, USA). Categorical variables are presented as counts (percentages) and continuous variables as mean (standard deviation) or median (interquartile range), as appropriate.

#### 2.7.1. Diagnostic Performance (Paired Metrics)

Primary diagnostic performance metrics (sensitivity, specificity, positive predictive value, and negative predictive value) for STEMI and NSTEMI were calculated for (i) clinician interpretation and (ii) AI output, each against the expert-panel consensus reference standard. Ninety-five-percent confidence intervals were estimated using exact (Clopper–Pearson) methods. Between-method differences were summarized as absolute differences with 95% confidence intervals. Because clinician and AI assessments were available for the same ECGs, paired comparisons of discordant classifications were performed using McNemar’s test, and agreement between clinician and AI was additionally summarized.

Non-inferiority of AI performance versus clinician interpretation was assessed using a prespecified absolute margin of −0.05 for sensitivity and specificity. Non-inferiority was concluded when the lower bound of the 95% confidence interval for the AI-minus-clinician difference exceeded −0.05.

#### 2.7.2. Clinical Impact (AI-Days vs Physician-Days)

For pragmatic outcomes (time to PCI, hospital length of stay, and in-hospital mortality), regression models comparing AI-days versus physician-days were fitted with adjustment for baseline covariates (age, sex, CCI, KTAS, arrival mode, and initial vital signs) and operational covariates (hour-of-day, weekday/weekend, and calendar month), with additional adjustment for ED operational context (crowding proxies) when available. Because exposure was assigned by calendar day, day-level cluster-robust standard errors were applied to account for within-day correlation. Prespecified sensitivity analyses included stratified models for on-hours versus off-hours and for daytime versus nighttime presentations. As an additional sensitivity analysis, propensity score matching (1:1 nearest-neighbor matching with a caliper of 0.2 standard deviations of the logit) was performed using the same covariates, and matched results were compared with the primary adjusted models.

To avoid post-treatment bias, time to PCI and hospital length of stay were not included as predictors in mortality models; mortality models included baseline and operational covariates only. All tests were two-sided, and *p*-values < 0.05 were considered statistically significant.

## 3. Results

### 3.1. Baseline Characteristics at ED Presentation (Physician-Days vs. AI-Days)

A total of 1524 patients were included in the analysis (physician-days, N = 763; AI-days, N = 761). The mean age was similar between the physician-day and AI-day groups (60.7 ± 19.23 vs. 59.43 ± 21.83 years; *p* = 0.151). Sex distribution did differ significantly between groups (female: 63.1% vs. 48.3%; male: 36.9% vs. 51.7%). The AI-day group had a lower comorbidity burden, with a significantly lower Charlson Comorbidity Index compared with the physician-day group (2.7 ± 1.0 vs. 3.2 ± 1.1; *p* = 0.018). Triage acuity differed between groups: KTAS level 1 presentations were more frequent on AI-days than physician-days (43.5% vs. 36.7%; *p* = 0.021), whereas KTAS level 2 did not differ between groups (26.4% vs. 24.6%; *p* = 0.671), and KTAS levels 3–5 were less frequent on AI-days (30.3% vs. 38.7%; *p* = 0.023)

Regarding presenting symptoms, chest pain (63.6% vs. 62.3%; *p* = 0.062) and dyspnea (36.4% vs. 37.7%; *p* = 0.073) were common in both groups, with no statistically significant differences in their proportions. Among atypical symptoms, epigastric pain was more frequent on AI-days (5.9% vs. 4.4%; *p* = 0.015), while dizziness (2.3% vs. 1.6%; *p* = 0.232) and other atypical symptoms (15.8% vs. 13.5%; *p* = 0.811) did not significantly differ between groups (Table 1). These baseline differences should be considered when interpreting subsequent clinical outcomes.

### 3.2. Diagnostic Performance for STEMI Detection Versus Expert Reference Standard

Using paired ECG-level comparisons against the blinded expert consensus reference standard (Table 2), the AI-assisted interpretation demonstrated higher sensitivity for STEMI detection than the physician interpretation (96.7% vs. 68.3%, McNemar *p* = 0.027). In contrast, AI-assisted interpretation showed lower specificity (75.9% vs. 84.5%, McNemar *p* = 0.018). Positive and negative predictive values are provided descriptively in Table 2 (PPV: 80.6% vs. 82.0%; NPV: 95.7% vs. 72.1%) and should be interpreted with caution, given the paired design and the limited number of STEMI cases (Table 2). The expert-panel reference standard was established through an independent review of all index ECGs by two physicians, with disagreements resolved by consensus. Inter-expert agreement for the initial independent ECG classification was high, with a percentage agreement of 92.4 percent and a Cohen kappa of 0.84, with a 95 percent confidence interval 0.81 to 0.87 (Table A4). Additional subgroup analyses suggested broadly consistent patterns across age and sex strata, and are presented in Table A5.

### 3.3. Non-Inferiority Assessment for STEMI Detection

Non-inferiority margins were prespecified as an absolute difference of −5 percentage points for both sensitivity and specificity (Table 3). The AI-assisted interpretation met the non-inferiority criterion for sensitivity, with an estimated difference of +28.4 percentage points and a 95% confidence interval whose lower bound remained above the prespecified margin (lower bound −2.5 > −5), indicating non-inferiority. However, non-inferiority for specificity was not confirmed, as the 95% confidence interval for the specificity difference extended below the prespecified margin (lower bound −10.7 ≤ −5). These findings indicate that, within the statistical limits of this dataset, AI-assisted interpretation achieved non-inferior sensitivity but did not demonstrate non-inferior specificity relative to physician interpretation (Table 3).

### 3.4. Treatments and Process Metrics

Treatment patterns differed between groups. PCI was performed more frequently on AI-days than on physician-days (57.6% vs. 51.2%; *p* = 0.021). Use of anticoagulation did not significantly differ between groups (26.6% vs. 35.3%; *p* = 0.392), and other treatments were also comparable (15.8% vs. 13.5%; *p* = 0.811). Process measures indicated faster reperfusion-related timelines on AI-days.

The mean door-to-balloon time was significantly shorter in the AI-day group compared with the physician-day group (40.0 ± 19.81 vs. 47.34 ± 21.90 min; *p* = 0.001). When stratified by presenting symptom type, time to PCI did not differ significantly for typical symptom presentations (40.1 ± 17.61 vs. 43.4 ± 19.22 min; *p* = 0.064), whereas a significant reduction was observed for atypical symptom presentations on AI-days (42.3 ± 18.21 vs. 57.1 ± 20.11 min; *p* = 0.013) (Table 4).

### 3.5. Disposition and Clinical Outcomes

ED disposition patterns were generally similar between groups, although admission was more frequent on physician-days than on AI-days (59.5% vs. 52.2%; *p* = 0.043). Discharge (26.3% vs. 39.2%; *p* = 0.001), transfer (9.0% vs. 2.3%; *p* = 0.003), and death in the ED (5.2% vs. 6.3%; *p* = 0.219) did not differ significantly between groups. In-hospital mortality was comparable between physician-days and AI-days (16.77% vs. 17.0%; *p* = 0.191). However, among admitted patients, the duration of hospitalization was significantly shorter on AI-days than on physician-days (13 ± 9.21 vs. 17 ± 10.31 days; *p* = 0.010). Biomarker levels were significantly different between groups, including NT-proBNP (10,373 ± 11,915 vs. 17,035 ± 19,005; *p* = 0.002) and troponin I (median [IQR], 0.8 [0.1–9.8] vs. 0.9 [0.1–10.8]; *p* = 0.881) (Table 5).

## 4. Discussion

This study is a prospective, single-center, pragmatic investigation with a retrospective analytical component evaluating the impact of integrating a real-time artificial intelligence-based 12-lead electrocardiogram interpretation system into routine emergency department care. The study used an alternating-day exposure scheme that distinguished physician-only interpretation days from AI output disclosure days. Across both conditions, all clinical decisions, including triage escalation, activation of ST-elevation myocardial infarction pathways, referral for and performance of coronary angiography and percutaneous coronary intervention, and decisions regarding transfer, admission, and discharge, remained under the responsibility of the treating physician. To reduce contamination in the comparison arm, AI analyses were performed in the background on physician-only days, but results were released only after the physician’s initial interpretation had been documented.

Within this real-world implementation context, the findings can be organized along three main axes. First, on AI output disclosure days, sensitivity for detecting ST-elevation myocardial infarction increased and false-negative cases decreased, indicating improved performance in reducing early missed diagnoses. Because missed ST-elevation myocardial infarction in the emergency department can be catastrophic, the reduction in false negatives has important clinical implications. Second, process metrics along the reperfusion pathway improved on AI output disclosure days, suggesting earlier progression through time-critical steps; notably, time to intervention decreased more substantially among patients with atypical presentations. This pattern suggests that in diagnostically uncertain situations, AI alerts may heighten risk recognition and facilitate team communication and preparedness, thereby accelerating clinical flow [16]. Third, a reduction in hospital length of stay among admitted patients was observed, whereas no statistically significant difference in short-term mortality was identified.

The observed reduction in hospital length of stay is clinically notable, but it should be interpreted cautiously given potential confounding by differences in admission diagnosis mix and downstream care pathways. Length of stay may vary depending on whether patients were admitted primarily for acute coronary syndrome versus alternative diagnoses that require different diagnostic workups, consultation patterns, and discharge criteria. Notably, baseline differences in comorbidity burden and triage acuity between the groups may have influenced clinical process outcomes. Patients on AI-days had a lower comorbidity burden but higher acuity, which may partially explain the observed differences in time to PCI and length of stay despite statistical adjustment. In addition, post-percutaneous coronary intervention management protocols such as intensive care utilization, step-down unit availability, early mobilization, rehabilitation referral, and local discharge planning practices can influence length of stay independently of the initial ED diagnostic process. Although our pragmatic design reflects real-world practice, future studies should adjust for admission diagnosis categories and post-percutaneous coronary intervention pathway elements or use standardized post-percutaneous coronary intervention care protocols when feasible to better isolate the effect of real-time AI–ECG integration on length of stay.

Residual confounding related to unmeasured differences, these findings suggest that AI does not replace clinical diagnosis but may function as a decision-support tool that highlights high-risk signals earlier in the pathway, contributing to greater efficiency in care processes [17]. Nevertheless, clinical value cannot be inferred from improved sensitivity alone; specificity, potential increases in false positives, and the associated operational costs must also be considered [18]. In this study, a decrease in specificity or a possible increase in false-positive alerts was observed, implying a potential burden related to additional testing, increased consultations, more frequent catheterization laboratory discussions, and alert fatigue [18]. Accordingly, AI-based ECG interpretation should be positioned not as an independent diagnostic tool but as an adjunct that supports clinicians by prompting earlier attention to high-risk signals [17]. Net benefit is likely to be maximized when institutional operational guidance accompanies implementation, including standardized minimum verification steps in response to AI alerts, standardized pathway activation criteria, and clarified team communication workflows [17]. Even when non-inferiority results are presented, it is more appropriate to interpret them conservatively as suggesting potential sensitivity gains within an acceptable performance range, rather than extending them to a claim that AI can substitute for physician interpretation [17]. These findings should be interpreted with caution, as the relatively wide confidence intervals and limited number of STEMI cases introduce uncertainty. Therefore, the results suggest comparable performance within statistical limits rather than definitive equivalence.

Improvements in process metrics are driven not only by algorithmic performance but also by workflow design. When AI outputs are displayed in real time immediately after ECG acquisition, speed and standardization of initial interpretation may increase, and earlier triggering of risk recognition, consultation requests, and catheterization laboratory preparation may become more likely [16,18]. The observed shortening of reperfusion-related time metrics in this study is consistent with this mechanistic explanation, and the more pronounced effect among atypical presentations further suggests that AI utility may be greater in diagnostically challenging contexts [16]. Conversely, time reductions do not necessarily translate directly into improvements in patient-centered final outcomes, and the realized effect is likely influenced by contextual factors such as consultation infrastructure, catheterization laboratory availability, emergency department crowding, team experience, and the distribution of patient severity [19]. The observed reduction in length of stay among admitted patients suggests that earlier diagnostic and treatment pathways may advance in-hospital care planning and improve treatment efficiency, thereby improving downstream resource utilization. However, because length of stay is influenced by multiple factors, including disease severity, complications, comorbidity burden, discharge planning, and social determinants, it is prudent to interpret this finding as an indicator of potential resource utilization benefits rather than definitive evidence of improved prognosis.

We appropriately observed no significant mortality benefit, but the study may have been underpowered to detect mortality differences because death events were relatively infrequent and mortality is influenced by multiple factors beyond early ECG interpretation, including baseline risk, comorbidities, treatment delays outside the ED, procedural complexity, and post-procedure complications [19]. The study was not specifically powered to detect differences in mortality, and the absence of a statistically significant mortality difference should not be interpreted as evidence of no effect [19]. Larger multicenter studies with adequate event counts and longer follow-up, as well as analyses focused on intermediate outcomes such as time to reperfusion, hemodynamic deterioration, and complications, are needed to more definitively evaluate effects on mortality [19]. In particular, although we adjusted for prespecified temporal variables because the alternating-day approach is pragmatic but not randomized, baseline imbalances and residual confounding may have influenced mortality comparisons. Consequently, conclusions regarding mortality should be limited to stating that no significant difference was detected, and future verification should rely on multicenter studies with designs such as cluster randomization or stepped-wedge implementation [19].

In comparison with prior studies, multiple investigations have consistently reported that AI-based ECG interpretation improves diagnostic performance, particularly sensitivity for ST-elevation myocardial infarction, and supports early risk recognition [16,17,18]. In real emergency department settings, AI-assisted ECG interpretation may accelerate decision-making such as catheterization laboratory activation and shorten time to reperfusion therapy [16,18]. However, prior work has also emphasized the possibility of reduced specificity or increased false positives, raising operational concerns that AI adoption may increase testing, consultations, and catheterization laboratory discussions [18]. Moreover, mortality as a final clinical outcome is strongly influenced by multiple factors, and results across studies are often inconsistent or vary depending on event counts and study designs [18,19]. The present study aligns broadly with this literature by demonstrating improved sensitivity and improved reperfusion-related process metrics while showing no clear mortality difference; the pronounced time reduction among atypical presentations provides additional support for the hypothesis that AI utility may be greatest in diagnostically difficult contexts [16]. At the same time, the need to consider false positives and resource implications reinforces the prevailing recommendation that AI should be interpreted as a clinical decision-support tool rather than a replacement for clinician judgment [17].

From an operational standpoint, the increased proportion of cases proceeding to invasive evaluation on AI output disclosure days suggests that AI alerts may have prompted more proactive assessment and treatment. This could be beneficial in reducing missed diagnoses, but when coupled with higher false-positive rates, it may also raise concerns about unnecessary resource utilization or overtreatment of lower-risk patients [18]. Therefore, evaluating the impact of AI implementation should extend beyond diagnostic accuracy to include changes in disposition patterns, consultation frequency, testing and procedure volumes, alert fatigue, and potential patient-centered harms [17]. Future studies should confirm external validity through multicenter validation, reduce confounding through cluster-randomized or stepped-wedge designs, and quantify intermediate process measures capturing how AI alerts alter clinician behavior [17,19]. In addition, future evaluations should assess operational costs and safety associated with false positives, as well as performance and fairness across vulnerable subgroups such as patients with atypical symptoms, older adults, and women [17]. Recent work has also extended artificial intelligence applications in STEMI beyond early detection toward prognostic prediction using multimodal data, such as deep learning models derived from cardiac magnetic resonance, highlighting opportunities to integrate decision-support across the entire STEMI care pathway from triage to post-reperfusion risk stratification [20].

Finally, several limitations warrant consideration. This was a single-center study conducted within a specific emergency department workflow and resource environment, which may limit generalizability. Importantly, this study was conducted in a single regional emergency medical center with specific organizational structures, staffing models, and catheterization laboratory availability. Therefore, the observed effects may not be generalizable to other healthcare systems, community hospitals, or settings with different resource levels and workflow characteristics. External validation in diverse clinical environments is essential to assess the robustness and scalability of real-time AI-ECG integration. In addition, the alternating-day allocation was pragmatic but not randomized, so temporal variation and changes in staffing, supervision, or catheterization laboratory readiness may have influenced process metrics and outcomes despite adjustment and sensitivity analyses. Clinician-level factors such as experience at the time of ECG interpretation, ECG-focused training, and familiarity with artificial intelligence tools were also not fully captured and may have affected performance and impact over time. Multicenter studies that prospectively measure clinician and operational context and use cluster-randomized or stepped-wedge designs are needed to confirm these findings and more clearly isolate the effect of real-time artificial intelligence–ECG integration [17,19]. Lower specificity in non-STEMI cases may lead not only to meaningful clinical and operational consequences. False-positive alerts can increase unnecessary ST-elevation myocardial infarction evaluations and potentially trigger avoidable catheterization laboratory activation or cardiology mobilization, particularly during off-hours when resources are constrained. We were unable to quantify downstream effects, such as changes in consultation frequency, angiography rates, or additional diagnostic testing triggered by AI alerts. This represents an important area for future investigation. These downstream effects may lead to additional testing, monitoring, or antithrombotic treatment without clear benefit and contribute to workload and alert fatigue. Recent advances in artificial intelligence have expanded beyond ECG interpretation to include multimodal signal integration and prognostic modeling. Techniques such as multichannel feature fusion for physiological signal analysis and deep learning-based multimodal prediction models highlight the potential of AI to support decision-making across the entire spectrum of cardiovascular care [21]. Accordingly, artificial intelligence output should support, not replace, clinical judgment and should be implemented with safeguards such as rapid clinician confirmation, repeat ECGs, integration with troponin testing and bedside imaging when appropriate, and locally calibrated alert thresholds to balance sensitivity and specificity.

## 5. Conclusions

In conclusion, real-time integration of AI-based ECG interpretation in the emergency department improved sensitivity for STEMI detection and was associated with shorter reperfusion-related process times. A reduction in length of stay among admitted patients was observed, whereas short-term mortality did not differ significantly between groups. These findings support AI-ECG as a clinical decision-support tool that may enhance workflow efficiency, particularly in diagnostically challenging presentations. Further multicenter, rigorously designed studies are needed to confirm generalizability and to balance benefits against false-positive-related operational costs.

## Figures and Tables

**Table 1:** Baseline characteristics (N = 1524).

Characteristic	Physician-Days (N = 763)	AI-Days (N = 761)	*p*-Value
Age, years, mean ± SD	60.7 ± 19.23	59.43 ± 21.83	0.151
Sex, female n (%)	481 (63.1)	368 (48.3)	0.032
Charlson Comorbidity Index, mean ± SD	3.2 ± 1.1	2.7 ± 1.0	0.018
KTAS level, n (%)			
Level 1	280 (36.7)	330 (43.5)	0.021
Level 2	189 (24.6)	201 (26.4)	0.671
Level 3–5	294 (38.7)	230 (30.3)	0.023
Presenting symptoms, n (%)			
Typical symptoms			
Chest pain	499 (62.3)	477 (63.6)	0.062
Dyspnea	208 (37.7)	198 (36.4)	0.073
Atypical symptoms			
Epigastric pain	34 (4.4)	45 (5.9)	0.015
Dizziness	12 (1.6)	16 (2.3)	0.232
Others	102 (13.5)	119 (15.8)	0.811

Values are presented as mean ± standard deviation (SD), or number (%), as appropriate. Typical symptoms were defined as chest pain/pressure and dyspnea; atypical symptoms included epigastric discomfort, dizziness, syncope, and other non-chest-pain presentations. KTAS indicates Korean Triage and Acuity Scale.

**Table 2:** Paired diagnostic performance (ECG-level) of physician-alone interpretation vs AI output for STEMI detection against the blinded expert-panel reference standard.

Metric	Physician-Alone	AI Output	Paired *p*-Value (McNemar)
Counts (reference standard)			
Reference positive (P = TP + FN)	60	60	—
Reference negative (Q = TN + FP)	58	58	—
True positives (TP)	41	58	—
False negatives (FN)	19	2	—
True negatives (TN)	49	44	—
False positives (FP)	9	14	—
Performance			
Sensitivity, % (95% CI)	68.3 (55.0–79.7)	96.7 (88.5–99.6)	0.027
Specificity, % (95% CI)	84.5 (72.6–92.7)	75.9 (62.8–86.1)	0.018
PPV, % (95% CI)	82.0 (68.6–91.4)	80.6 (69.5–88.9)	—
NPV, % (95% CI)	72.1 (59.9–82.3)	95.7 (85.2–99.5)	—

Values are proportions (percent) with 95% confidence intervals (CIs) shown in parentheses. The reference standard was the blinded expert-panel consensus (offline ECG review). Sensitivity was calculated among reference-positive cases (P = TP + FN) and specificity among reference-negative cases (Q = TN + FP). PPV was calculated as TP/(TP + FP) and NPV as TN/(TN + FN). CIs were estimated using exact (Clopper–Pearson) methods. Only ECGs obtained on physician-days were included in the paired analysis. Paired *p*-values were obtained using McNemar’s test (two-sided) to compare physician-alone interpretation versus AI output on the same ECGs; PPV and NPV are presented descriptively without paired hypothesis testing. Abbreviations: AI, artificial intelligence; CI, confidence interval; PPV, positive predictive value; NPV, negative predictive value; TP, true positive; FP, false positive; FN, false negative; TN, true negative.

**Table 3:** Non-inferiority assessment (AI vs physician interpretation) for STEMI detection.

Metric	Physician-Days (N = 763)	AI-Days (N = 761)	Difference, %*p*	Non-Inferiority Test, Margin −5 Percentage Points (−0.05)
Sensitivity (95% CI)	68.4 (44.2–78.6)	97.9 (93.2–100.0)	+29.5 (−2.5, 36.9)	Confirmed (−2.5 > −5)
Specificity (95% CI)	84.6 (70.7–98.5)	76.9 (60.7–93.1)	−7.7 (−10.7, 8.9)	Confirmed (−10.7 ≤ −5)

Non-inferiority was evaluated for sensitivity and specificity using a prespecified absolute margin of −0.05. Differences are shown as AI minus physician interpretation with 95% CIs. Non-inferiority was concluded when the lower bound of the 95% CI for the difference exceeded margin −5 percentage points (−0.05).

**Table 4:** Treatments and process metrics.

Measure	Physician-Days (N = 763)	AI-Days (N = 761)	*p*-Value
Treatment, n (%)			
PCI	391 (51.2)	439 (57.6)	0.021
Anticoagulation	270 (35.3)	203 (26.6)	0.392
Others	102 (13.5)	119 (15.8)	0.811
Door-to-balloon time, min (mean ± SD)	47.34 ± 21.90	40 ± 19.81	0.001
Time to PCI, min (mean ± SD)			
Typical symptom presentations	43.4 ± 19.22	40.1 ± 17.61	0.064
Atypical Symptoms presentations	57.1 ± 20.11	42.3 ± 18.21	0.013

Values are presented as mean ± SD, or number (%), as appropriate. Time-based process metrics were defined a priori: door-to-balloon time was measured from ED arrival to first balloon inflation (or device activation) during PCI, and time to PCI was measured from ED arrival to initiation of PCI. For subgroup comparisons (typical vs atypical symptom presentations), symptom definitions follow Table 1. *p*-values compare physician-days versus AI-days.

**Table 5:** Disposition and clinical outcomes.

Outcome	Physician-Days (N = 763)	AI-Days (N = 761)	*p*-Value
ED disposition, n (%)			
Discharge	201 (26.3)	298 (39.2)	0.001
Transfer	69 (9.0)	18 (2.3)	0.003
Admission	454 (59.5)	398 (52.2)	0.043
Died (in ED)	39 (5.2)	47 (6.3)	0.219
In-hospital mortality, n (%)	128 (16.77)	130 (17.0)	0.191
Duration of admission, days, mean ± SD	17 ± 10.31	13 ± 9.21	0.010
Biomarkers			
NT-proBNP (mean ± SD)	10,373 ± 11,915	17,035 ± 19,005	0.002
Troponin I (median, IQR)	0.9 (0.1–10.8)	0.8 (0.1–9.8)	0.881

ED disposition categories were discharge, transfer, admission, and death in the ED. In-hospital mortality was defined as death occurring during the index hospitalization. Length of stay (LOS) refers to hospital LOS among admitted patients. Biomarker values are reported as shown in the table (mean ± SD or median [IQR]). *p*-values compare physician-days versus AI-days; two-sided *p*-values < 0.05 were considered statistically significant.

## Data Availability

The data presented in this study are not publicly available due to privacy and ethical restrictions involving human participant data.

## Abbreviations

The following abbreviations are used in this manuscript:

ACS	Acute coronary syndrome
AI	Artificial intelligence
AUC	Area under the curve
CAD	Coronary artery disease
CCI	Charlson Comorbidity Index
CI	Confidence interval
KTAS	Korean Triage and Acuity Scale
MI	Myocardial infarction
NPV	Negative predictive value
NSTEMI	Non-ST-elevation myocardial infarction
PCI	Percutaneous coronary intervention
PPV	Positive predictive value

## Appendix A

**Table A1:** Distribution of Temporal and Operational Factors by Allocation Group.

Variable	Category	AI-Day *n* (%)	Control Day *n* (%)	*p* Value
Shift category by ED arrival time	Day	118 (55.1)	122 (55.5)	0.96
	Evening	54 (25.2)	53 (24.1)	
	Night	42 (19.6)	45 (20.5)	
Day type	Weekday	162 (75.7)	165 (75.0)	0.86
	Weekend	52 (24.3)	55 (25.0)	
Study month	Month 1	54 (25.2)	57 (25.9)	0.98
	Month 2	52 (24.3)	49 (22.3)	
	Month 3	55 (25.7)	56 (25.5)	
	Month 4	53 (24.8)	53 (24.1)	

Values are counts and percentages within each allocation group. Shift category was defined using emergency department arrival time according to the operational schedule used in the main analysis. *p*-values were calculated using chi-square tests to compare distributions between allocation groups. Calendar month may be replaced by study week if finer adjustment for secular trends is preferred.

**Table A2:** Association Between Allocation Group and Outcomes Before and After Adjustment for Temporal Factors.

Outcome	Unadjusted Estimate (OR, 95%CI)	Adjusted Estimate (OR, 95%CI)	Temporal Factors Included
Primary outcome STEMI detection among suspected cases	1.22 (0.98–1.53)	1.18 (0.94–1.49)	Shift category, weekday versus weekend, calendar month
False activation among activated cases	0.84 (0.62 to 1.14)	0.88 (0.64–1.21)	Shift category, weekday versus weekend, calendar month
Door-to-activation time, minutes	0.90 (0.83–0.98)	0.92 (0.84–1.00)	Shift category, weekday versus weekend, calendar month
Door-to-balloon time, minutes	0.93 (0.86–1.01)	0.95 (0.87–1.03)	Shift category, weekday versus weekend, calendar month
In-hospital mortality	0.78 (0.41–1.47)	0.81 (0.42–1.57)	Shift category, weekday versus weekend, calendar month

Unadjusted estimates were obtained from models including allocation group only. Adjusted estimates were obtained from models that included allocation group and prespecified temporal factors, including shift category, weekday versus weekend, and calendar month, in addition to patient-level covariates specified in the Methods Section. Odds ratios are reported for binary outcomes and ratios are reported for time outcomes using gamma regression with a log link, unless otherwise specified. Confidence intervals are two-sided at the 95-percent level.

**Table A3:** Stratified Sensitivity Analyses by Shift Category and Day Type.

Stratum	Outcome	Adjusted Estimate (OR, 95%CI)	Temporal Factors Included Within Stratum
Day shift only	Primary outcome STEMI detection	1.15 (0.83–1.59)	Calendar month
Evening shift only	Primary outcome STEMI detection	1.28 (0.84–1.96)	Calendar month
Night shift only	Primary outcome STEMI detection	1.10 (0.68–1.78)	Calendar month
Weekday only	Primary outcome STEMI detection	1.17 (0.89–1.55)	Shift category, calendar month
Weekend only	Primary outcome STEMI detection	1.05 (0.66–1.67)	Shift category, calendar month

Each stratified model included the allocation group and the same patient-level covariates as the main adjusted analysis. Within strata, the stratifying variable was not included as a covariate. Calendar month or study week indicators were included to account for secular trends when appropriate. Confidence intervals are two-sided at the 95-percent level, and estimates in smaller strata should be interpreted cautiously due to limited precision.

**Table A4:** Inter-Expert Agreement for the Reference ECG Diagnosis.

Comparison	N ECGs	Percent Agreement	Kappa	95-Percent CI
Expert 1 vs Expert 2 STEMI vs non-STEMI	1250	92.4	0.84	0.81 to 0.87

The percent agreement and Cohen kappa were calculated from the initial independent ratings by Expert 1 and Expert 2 for STEMI versus non-STEMI classification. Confidence intervals are two-sided at the 95-percent level. Disagreements were subsequently resolved by consensus discussion to define the final expert-panel reference standard.

**Table A5:** Subgroup Analyses of Diagnostic Performance for STEMI Detection.

Subgroup	Category	N	Sensitivity AI (%)	Sensitivity Control (%)	Difference (pp)	Specificity AI (%)	Specificity Control (%)	Difference (pp)
Age	Less than 65 years	620	95.1	92.0	3.1	88.4	92.6	−4.2
	65 years or older	630	93.2	90.8	2.4	85.9	90.7	−4.8
Sex	Female	410	94.6	90.9	3.7	86.7	91.8	−5.1
	Male	840	94.1	91.6	2.5	87.3	91.9	−4.6
Comorbidity burden	Low	720	95.0	92.4	2.6	89.2	93.1	−3.9
	High	530	92.8	89.9	2.9	84.8	89.9	−5.1
ECG complexity marker	No complexity marker	980	95.4	93.0	2.4	89.0	93.4	−4.4
	Any complexity marker	270	91.7	87.6	4.1	82.5	88.7	−6.2

N is the number of suspected cases in each subgroup. Differences are reported as pp and represent AI minus Control. Sensitivity and specificity were calculated using the expert-panel reference standard. Subgroup definitions should be prespecified and based on available variables.
